# A Review on the Role of Vibrational Spectroscopy as An Analytical Method to Measure Starch Biochemical and Biophysical Properties in Cereals and Starchy Foods

**DOI:** 10.3390/foods3040605

**Published:** 2014-12-01

**Authors:** D. Cozzolino, S. Degner, J. Eglinton

**Affiliations:** School of Agriculture, Food and Wine, The University of Adelaide, Waite Campus, PMB 1 Glen Osmond, SA, 5064, Australia; E-Mails: sophia.degner@adelaide.edu.au (S.D.); jason.eglinton@adelaide.edu.au (J.E.)

**Keywords:** gelatinization, pasting properties, starch, near infrared spectroscopy, mid infrared spectroscopy

## Abstract

Starch is the major component of cereal grains and starchy foods, and changes in its biophysical and biochemical properties (e.g., amylose, amylopectin, pasting, gelatinization, viscosity) will have a direct effect on its end use properties (e.g., bread, malt, polymers). The use of rapid and non-destructive methods to study and monitor starch properties, such as gelatinization, retrogradation, water absorption in cereals and starchy foods, is of great interest in order to improve and assess their quality. In recent years, near infrared reflectance (NIR) and mid infrared (MIR) spectroscopy have been explored to predict several quality parameters, such as those generated by instrumental methods commonly used in routine analysis like the rapid visco analyser (RVA) or viscometers. In this review, applications of both NIR and MIR spectroscopy to measure and monitor starch biochemical (amylose, amylopectin, starch) and biophysical properties (e.g., pasting properties) will be presented and discussed.

## 1. Introduction

The starch stored in the seeds and tubers of various agricultural crops including maize, wheat, rice, barley, potato and cassava provides the main source of energy in the human diet [1,2,3,4]. Starch is the major component of cereal grains, and changes in its biophysical and biochemical properties are related with the amount and ratio of amylose and amylopectin, that influence and affect properties such as viscosity, gelatinization, that will determine its end use properties (e.g., bread, malt, beer, polymers) [1,4,5,6,7].

Current chemical and physical methods used in research and by the industry to measure starch properties are slow, destructive, with many based on empirical relationships [8,9,10,11,12]. Overall knowledge of the pasting properties of the sample can help to improve starch content in cereals and starchy foods as well as will allow understanding the biophysical and structural properties of the starch to be used in foods (e.g., bread, beer, whisky) and in industrial applications (e.g., polymer production) [13]. This can also provide with useful information about the specific application of the starch in the selection or screening of new genotypes or lines in breeding programs [6,14].

Analytical methods currently used to determine biophysical properties in cereals and starchy foods include instrumental methods such as Differential Scanning Calorimetry (DSC) and the Rapid Visco Analyzer (RVA) [1,9,10,11,13,15,16,17]. Instruments such as the RVA are also used in the routine analysis of cereals (e.g., wheat, barley) to determine the effects of rain damage on grain quality at the delivery point [17,18,19].

During the last 20 years, methods based on vibrational spectroscopy in combination with chemometric techniques have resulted in the development of rapid methods to predict and monitor starch biochemical and biophysical properties [8,9,10,11]. Desirable characteristics of these tools include speed, ease-of-use, minimal or no sample preparation, and in some case the avoidance of sample destruction. Most of these features are characteristic of mid-infrared (MIR) and near-infrared (NIR) spectroscopy [8].

In a recent report, Kaddour and Cuq [20,21] highlighted three main ways in which NIR spectroscopy is currently applied in the analysis of cereals and starchy products: (1) as the straightforward analysis and very rapid determination of composition; (2) as screening tool in plant-breeding (for the selection of cross-breeds with the desired qualities); and (3) as an in-line tool to monitor physical and chemical changes during processing.

In this review, applications of both NIR and MIR spectroscopy to measure and monitor starch biochemical composition (e.g., amylose, amylopectin and starch) and biophysical properties (e.g., pasting properties, viscosity) will be presented and discussed.

## 2. Applications of NIR and MIR Spectroscopy

Several authors reported the ability of both NIR and MIR spectroscopy to measure and monitor starch biochemical and biophysical characteristics in a wide range of starchy foods and cereals. Examples of these applications are discussed and summarised below.

### 2.1. Determination of Amylose, Amylopectin, Starch and Granule Structure

The feasibility of using NIR reflectance spectroscopy to analyse starch and amylose, in buckwheat flours was reported [22]. Samples from different cultivars of buckwheat harvested in 12 different countries were analysed using NIR and multiple linear regression (MLR). The authors reported correlation coefficients (R) higher than 0.93 for starch, while workable standard error of predictions (SEP) in relation to the reference standard deviation data were reported. In contrast, the MLR models for amylose were judged as unstable due to the poor SEP values obtained. These authors attributed this issue to the standard deviation of the reference data [22]. The main wavelengths reported by the authors were found around 1925 nm associated with water (combination of the stretching and bending vibrations of hydroxyl group), around 2057 nm associated with protein (combination of NH and amide II or III), and around 2100 nm associated with starch (combination of OH and CO) [22]. The authors concluded that NIR spectroscopy can only be used for the measurement of the amounts starch in buckwheat flours [22].

Near infrared analysis was used to predict starch, moisture, and sugar content in sliced and fresh samples of sweetpotato (*Ipomoea batatas* (L.) Lam.). Samples were collected during three growing seasons, where the best calibration equation for starch was developed from the combination of samples from the three harvests [23]. An *R* of 0.95, standard error of calibration (SEC) of 2.01, and SEP of 1.91 was reported by these authors [23] for the determination of starch content. Calibrations based on samples from a given year adequately predicted the variables but could not account for variances introduced by samples from other years or harvests [23].

The use of NIR transmittance (NIT) spectroscopy was assessed to measure amylose content in corn (*Zea mays*) [24]. Calibrations were developed using a set of genotypes having endosperm mutations in single and double-mutant combinations, ranging in starch-amylose content (SAC) from 8.5% to 76% [24]. Prediction models for SAC yield an *R* of 0.96 and SEP of 5.1% [24]. According to the authors, narrowing the amylose range of the calibration set generally did not improve performance statistics except when partial least squares regression (PLS) was used, in which a decrease in the SEP values were observed [24]. Although the amylose calibrations were of limited precision, the authors stated that the calibration models developed may be useful when a rough screening method is needed for SAC [24].

The determination of starch in potato tubers sourced from different varieties and breeding lines was achieved using NIR spectroscopy [25]. The authors reported an *R*^2^ of 0.90 and SECV values ranging between 0.74% and 0.79% [25,26]. Amylose content was measured in sorghum samples using differential scanning calorimetry and predicted using NIR spectroscopy [27]. The *R*^2^ and SECV for the determination of amylose in ground and whole sorghum grain were 0.75 (SECV = 0.77) and 0.70 (SECV = 0.75), respectively [27].

The potential of NIR reflectance spectroscopy was investigated as an alternative method for predicting the major constituents in yam tuber samples (*Dioscorea pp*) [28,29]. Two hundred and sixty-five samples, belonging to seven different yam accessions were analysed for starch, amylose, sugars, proteins, minerals and cellulose [28,29]. The reported coefficients of determination (*R*^2^) were 0.84 for starch, 0.86 for sugars and 0.88 for proteins [28,29]. Calibrations developed by combining both the calibration and validation sets determined an improvement of the calibration statistics [29]. Discriminant analysis (DA) was also used by the authors to classify samples according to the amylaceous fraction of the chemotype [29].

A methodology for the rapid estimation of taro quality (*Colocasia esculenta*) (starch, total sugars, cellulose, proteins, and minerals) was reported using NIR spectroscopy [30]. NIR calibration models reported a *R*^2^ of 0.89 for starch, 0.90 for sugars, 0.44 for amylose and 0.61 for cellulose [30]. The predictions were tested on an independent set of 58 randomly selected accessions and the *R*^2^ in prediction for starch and sugars were 0.76 and 0.74 respectively [30].

Wheat (*Triticum aestivum* L.) samples sourced from breeding programs that developed genotypes free of amylose (waxy wheat), as well as genetically intermediate (partial waxy) types were analysed using NIR spectroscopy [31,32,33,34]. Linear discriminant analysis (LDA) identifies the fully waxy genotype samples (greater than 90% accuracy) [34]. However, accuracy was reduced for partial and wild-type genotypes. It was suggested by the authors that the spectral sensitivity to waxiness is due to the lipid-amylose complex which diminishes with waxiness. These physical differences in endosperm affect light scatter as well as determine changes in starch crystallinity [34].

Interactions between carbohydrate monomers and polymers and their effects on NIR spectra were explored as well as the implications of such effects with regards to the development of NIR calibrations were evaluated [35]. The effects of the presence of amylopectin, amylose, cellulose and starch during the drying of glucose and sucrose on the resulting spectra were investigated [35]. Sugars in various molar ratios with polymers were dried in a rotating mixer and then reground, and spectra from 10,000 to 4000 cm^−1^ were recorded [35]. Although simple mixing of sugars with cellulose, amylose, amylopectin or starch caused some changes in the spectra of the sugars, in general the spectra obtained by spectral subtraction were considerably more like those of the pure sugars than were those obtained for the materials dried together [35].

The use of Fourier transform infrared (FTIR) spectroscopy was used as tool to differentiate between patterns of amylose in different granule types [36]. According to the authors, the IR spectrum of starch samples was described by peaks near 3500, 3000, 1600, 1400, 1000, 800 and 500 cm^−1^ [36]. The peaks at 3405 and 2930 cm^−1^ could be attributed to O–H and C–H bond stretching, respectively, while the peaks at 1420 and 1366 cm^−1^ were attributed to the bending modes of H–C–H, C–H and O–H. The peaks at 1300~1000 cm^−1^ were attributed to C–O–H stretching. The peaks at 1155, 1097 and 1019 cm^−1^ were assigned as the C–O bond stretching. The bands at 1047 and 1022 cm^−1^ were associated with the ordered and amorphous structures of starch, respectively. The ratio between absorbances 1047/1022 cm^−1^ was also used to quantify the degree of order in starch samples [36].

The structure of the A-and B-type granules of wheat starch was measured using polarized light microscopy, X-ray diffraction (XRD), and FTMIR spectroscopy in order to study the granular, crystalline, and short-range structures [37,38]. The A-and B-type granules displayed a typical A-type crystalline structure with the degrees of crystallinity of 31.95% and 29.38% respectively. A second order reflection was found in both A-and B-type granules, which was proposed due to the crystalline lamellae of the semicrystalline lamellae. The A-and B-type granules had mass and surface fractal structures respectively [37,38]. Table 1 shows the validation statistics for the analysis of amylose and starch content in different cereals and starchy products using NIR spectroscopy as well as different reference methods.

**Table 1:** Validation statistics for the measurement of amylose and starch, using near infrared spectroscopy and different reference analytical methods as reported by various authors.

Chemical parameter	Reference method	Sample	SECV/SEP	Reference
Amylose (%)	Iodine—colorimetric method	Rice	0.30–0.31	[39]
Amylose (g kg^−1^)	Iodine—colorimetric method	Beans ^1^	11.4–12.8	[40]
Amylose (%)	Enzymatic	Barley	0.93–1.09	[41]
Starch (%)	Enzymatic	Barley	0.78–0.98	[41]
Amylose (%)	Iodine—colorimetric method	Yam	3.71	[29]
Starch (%)	Iodine—colorimetric method	Yam	1.78	[29]
Crude starch (%)	Acetic acid-calcium chloride and polarization	Maize	0.72–0.96 *	[42]

Notes: SECV: standard error of cross validation; SEP: standard error of prediction; ^1^ handheld and bench instruments were compared; ^*^ RMSEP: root mean square error of prediction.

### 2.2. Gelatinization, Pasting Properties and Retrogradation of Starch

The first attempt to get information correlated to rice RVA pasting data by NIR spectroscopy was only partially successful and reported by Delwiche and collaborators [33]. Later few studies were found in the literature that attempted to characterise the biophysical properties of starchy samples (e.g., pasting properties, viscosity) using either NIR or MIR spectroscopy [43,44,45,46,47,48]. Calibration and validation statistics reported by other authors on the use of NIR spectroscopy to predict pasting properties in cereals are summarised in Table 2.

**Table 2:** Calibration and validation statistics for the determination of pasting properties using near infrared spectroscopy in different cereal and starchy samples as reported by several authors.

Sample	Method and wavelength range	Parameter (RVU)	*R*^2^	SECV/SEP	Reference
Rice	NIR (400–2500 nm)	PV	0.35	17.5	[49]
		BD	0.88	10.2	
		SB	0.92	13.6	
		HPV	0.55	16.7	
Rice non-waxy	NIR (1100–2500 nm)	PV	0.37	32.44	[50]
		BD	0.58	13.36	
		SB	0.60	25.07	
Rice	NIR (1100–2500 nm)	PV	0.63	23.7	[33]
		BD	0.72	14.2	
		SB	0.73	20.2	
Rice	NIR (1100–2500 nm)	PV	0.38–0.42		[43]
		BD	0.057–0.060		
		SB	0.57–0.59		
Rice	NIR (1100–2500 nm)	PV	0.74	20.99	[51]
		BD	0.80	21.47	
		SB	0.97	22.23	
		TH	0.80	7.37	
		FV	0.95	13.2	
Sweet potato	NIR (1100–2500 nm)	PV	0.91	13.1	[46,47]
		BD	0.81	10.67	
		SB	0.92	1.82	
Maize	NIR (1100–2500 nm)	PV	0.92	183	[44]
		BD	0.92	232	
		SB	0.92	412	

Notes: RVU: rapid visco units; *R*^2^: coefficient of determination; SECV: standard error of cross validation; SEP: standard error of prediction; PV: peak viscosity; BD: breakdown; SB: setback; HPV: hot pasting viscosity; TH: trough; FV: final viscosity.

The ability of NIR spectroscopy to analyse changes in structure of starch due to gelatinization, and determination of degree of gelatinization was reported. The second derivative NIR spectra of rice starch samples having different degrees of gelatinization, showed large deviations at wavelengths around 1204, 1368, 1436, 1700, 1748, 1784, 1924, 2088, 2280, 2320 and 2348 nm [52]. Especially, NIR spectra in the wavelength region around 2100 and 2280 nm changed complexly according to progress of gelatinization. According to the authors particle size effects could explain some of the high correlations obtained (high correlation existed between degree of gelatinization and particle size) [52].

The gelatinization of rice starch was reported as a function of temperature and pressure from the changes in the IR spectrum [53]. These authors proposed that the re-entrant shape for starch is not only due to hydrogen bonding but also to the imperfect packing of amylose and amylopectin chains in the starch granule [53].

The RVA instruments are widely used in assessing cooking and processing characteristics in rice [43]. The ability to predict RVA parameters by NIR spectroscopy would be useful in rapidly determining rice pasting qualities, but NIR spectroscopy does not correlate with the traditional parameters such as peak viscosity (PV), final viscosity (FV), breakdown (BD), consistency, and setback (SB) [43]. Alternative RVA parameters were sought by collecting RVA and NIR data for a total of 86 short, medium, and long grain rice cultivars. The amylose contents were 0.41%–24.90% (w/w) and protein concentrations were 8.47%–11.35% (w/w). PLS regression models generated for the entire NIR spectrum against the RVA curve showed viscosity varied linearly with the NIR spectra between 1100 to 2500 nm [43].

The use of NIR reflectance spectroscopy for the rapid and accurate measurement of starch gelatinization degree was explored in fresh pasta made with eggs [54,55]. The samples (*n* = 48) were analysed using a FT-NIR spectrophotometer [54,55]. Modified PLS (MPLS) model was developed to predict the gelatinization degree. The model was able to accurately predict gelatinization degree with SEP values of 0.24 and *R* of 0.97 [54,55].

Lu and co-workers [46,47] reported good calibration statistics using sweet potato. The results obtained in this study proved that predictions of parameters derived from the RVA by NIR spectroscopy are sufficiently accurate to be applied as indicators of quality in breeding, especially for early selection of materials in breeding [46,47,56]. However, because NIR spectroscopy is a secondary technique relying on calibration against a reference analysis (RVA profile), and the quality of the calibration process is critical, for accurate testing of advanced breeding lines, instrumental testing with RVA will still require for confirmation of differences in quality parameters [46,47,56]. Precision and reliability of NIR calibration and prediction may be affected by many factors, such as sample representativeness, genetic variability for traits, accuracy of reference data, as well as other factors [46,47]. The genetic variability available for some traits was the most limiting factor in achieving high *R* values. A rapid predictive method based on NIR spectroscopy was developed to measure sweet potato starch physiochemical quality and pasting properties [46,47]. The results reported by the authors showed that NIR analysis was sufficiently accurate and effective for rapid evaluation of starch physicochemical properties in sweet potato. According to the authors the NIR based protocol developed in this study can be used for screening large number of starch samples in food enterprises and sweet potato breeding programs [46,47].

Bao and collaborators [49] reported that the PV and HPV derived from the RVA profiles were poorly predicted, while BD and SB achieved better prediction with low SEP (<20.8 RVU; RVU, rapid visco units). The same authors also reported differences between NIR calibrations for RVA parameters when waxy and non-waxy rice samples were analysed [49,50].

Three RVA profile parameters of rice were predicted with NIR spectroscopy [57,58]. The coefficients of determination of calibration (*R*^2^) for BD, SB and CSV were 0.97, 0.99 and 0.99, respectively [58]. The root mean square errors of calibration (RMSEE) were 4.12, 2.41 and 1.72 [58]. The *R*^2^ of NIR models of brown rice RVA profile parameters was 0.94, 0.99 and 0.99, respectively. The RMSECV was 5.4, 2.87 and 1.99, respectively [58].

Calibration models based on NIR spectra were developed using modified PLS regression with different mathematical treatments based on the grain and flour spectra of non-waxy rice alone or in combination with waxy rice [50]. The results showed that calibration models built with flour spectra are more robust than those with grain spectra, and with total rice including waxy rice are superior to those with only non-waxy rice [50]. However, for accurate assay of the pasting viscosity and gel textural parameters, direct instrumental measurement should be employed in later generations [50]. Wu and collaborators [58,59] also reported the prediction of amylose content using NIR spectroscopy. They reported differences in the calibrations obtained using brown, whole and rice flour [58,59].

The performance of NIR spectroscopy as method was examined for the non-destructive determination of major components of potato (*Solanum tuberosum*) and its pasting viscosity properties [60]. Good calibration models with reasonable accuracy for moisture, carbohydrate, protein and amylase contents and RVA parameters were determined using PLS regression [60]. The SEP values obtained were 0.87% for moisture, 0.95% for carbohydrates, 0.6% for amylose, and 0.15% for protein, as well as 30 RVU for PV, 34 RVU for FV, 24 RVU for BD, and 22 RVU for ST. The calibration model for ash content was not significant; further research is needed to improve calibration for ash content [60]. Thus, NIR spectroscopy is a useful method to non-destructively measure the major components and pasting viscosity of intact potato [60].

Retrogradation of gelatinised starch is the main phenomenon that influences the texture of MiGao (rice cake) [61]. The hardness of the MiGao increased during stored at 25 °C for 5 days. The RVA, FTIR spectroscopy and X-ray were used in order to quantify and analyze the retrogradation behaviour of MiGao [61].

Three rice starches with different amylose contents (Glutinous: 1.4%, Jasmine: 15.0% and Chiang: 20.2%) were pregelatinized in a double drum dryer at 110, 117 and 123 °C. Starch crystallinity was determined by XRD and FTIR spectroscopy [62]. Rheological properties were assessed using RVA and a rheometer. Pre-gelatinized starches obtained from Glutinous (PGS) and Jasmine rice (PJS) gave an RVA pasting profile with cold PV [62].

Glutinous flour derived from rice varieties such as RD6, Sakonnakorn and Niew Ubon as well as Horn-Mali rice flour samples were analysed using both RVA and NIR spectroscopy [51]. The models for determining the pasting properties using the PT, ST, FV, BD and trough (TH) yield all had a similar level of accuracy with *R*^2^ values of 0.99, 0.97, 0.95, 0.80 and 0.80, respectively [51]. Furthermore, the thermal properties indicated that the models for onset temperature, peak temperature, and enthalpy produced moderate correlation values with *R*^2^ values of 0.82, 0.75 and 0.73, respectively, whereas pasting temperature could not be predicted by NIR spectroscopy (*R*^2^ = 0.06) [51].

Twenty-two diverse sorghum landraces, classified as normal and opaque types obtained from Ethiopia, were characterised for grain quality parameters using NIR spectroscopy, chemical and the RVA method [19]. Protein content ranged from 77 to 182 g kg^−1^, and starch content from 514 to 745 g kg^−1^. The normal sorghums had higher digestible energy than the opaque sorghums, which exhibited lower RVA viscosities, and higher pasting temperatures and ST ratios [19]. The RVA parameters were positively correlated with the starch content and negatively correlated with the protein content. Landraces were different for the various grain quality parameters with some landraces displaying unique RVA and NIR profiles [19]. The authors concluded that this study might guide utilisation of the sorghum landraces in plant improvement programs, and provides a basis for further studies into how starch and other constituents behave in and affect the properties of these landraces [19].

In recent years, the use of attenuated total reflectance and mid infrared (ATR-MIR) spectroscopy was evaluated and used to understand the gelatinization and retro-gradation of flour barley samples and the relationship with malting quality. Samples were sourced from commercial barley varieties, analysed using RVA and ATR-MIR instrument. These results showed that ATR-MIR spectroscopy is capable of characterising gel samples derived from barley flour samples having different malting characteristics. Infrared spectra can effectively represent a “fingerprint” of the sample being analysed and can be used to simplify and reduce analytical times in the routine methods currently used [11].

### 2.3. Monitoring of Starch Gelatinization and Processing

Currently a large proportion of analytical and routine process steps or controls in the cereal industry are performed in the laboratory. Vibrational spectroscopy have proven to be very versatile in dealing with either inorganic or organic constituents within a broad range from environmental to industrial applications [63,64]. The main advantages of these techniques are their multiplexing capability: more than ten different and locally separated measurement positions can be monitored with only one instrument, require minimal sampling; hence, in conjunction with chemometric modelling, it often replaces analytical methods like chromatography [63,64]. In recent years, the term process analytical technologies (PAT) describes the field of process analysis and measurement technologies that have been expanded to include several physical, chemical, mathematical and other analytical tools used to characterize chemical and biological processes [63,64,65]. The so called PAT technologies demonstrated to be one of the most efficient and advanced tools for continuous monitoring, as well as controlling the processes and the quality of raw ingredients and products in several applications among food processing, petrochemical and pharmaceutical industries [63,64,65].

According to Kaddour and collaborators [20,21] NIR spectroscopy is probably one of the physical methods best adapted to analyse starch products in cereals and starchy foods. The use of NIR spectroscopy as an in-line monitoring tool in cereal processing has opened a new field of applications, whereby NIR spectroscopy can provide information on changes in chemical and physico-chemical characteristics of foods during manufacturing processes and has been reported by various authors [65,66,67,68,69,70,71,72,73,74,75,76,77]. Recently, the use of dynamic NIR spectroscopy provides information that is relevant to calculate the reaction rate constants for the process [20,21,78,79,80]. Dynamic NIR spectroscopy gives complementary information with those that can be obtained using classical analytical methods, such as rheology or chemical analysis [20,21,78,79,80].

The potential applications of dynamic NIR spectroscopy for cereal products concern in-line monitoring of unit operations involved in transformation of wheat flour to food products (flour agglomeration, dough mixing, pasta dough extrusion and sheeting, dough proofing, thermal treatment and storage) [20,21,78,79,80]. The changes in NIR spectrum *versus* mixing time were considered to construct the NIR mixing curve and to determine the optimum mixing times. Several research groups [65,78,79,80,81,82,83,84,85] have also confirmed the ability of NIR spectroscopy to monitor dough mixing and extended applications over different mixer systems, in particular in wheat and dough formulations [20,21,78,79,80] as well as during potato processing [77]. The use of NIR technology in breadmaking process due to its non-invasive aspect would lead to both the industrial automation of the mixing process and the better description of physico-chemical phenomenon implied during dough mixing. A more detailed literature analysis on the NIR monitoring of bread dough mixing was presented recently in a review by Kaddour and Cuq [20,21].

The physicochemical events occurring during batter mixing at different water contents (51.8, 54.4, and 56.7 g of water/100 g of dough) were described and analysed using NIR spectroscopy [84]. An FT-NIR spectrometer over the 1000–2500 nm range with a fibre optic probe was used to record NIR spectra in-line. The analysis of both one-dimensional statistical method such as PCA and generalised two-dimensional correlation spectroscopy (2D COS) was conducted to evaluate the possibilities of NIR spectroscopy to monitor physical and physicochemical modifications observed during mixing of batter [84]. The NIR results were in agreement with the physical and physicochemical analysis traditionally used to study bread dough mixing including consistency and glutenin depolymerisation [84]. The 2D COS method allowed a sequence of chemical events occurring during mixing for the batters at 51.8% and 54.4% water contents to be tentatively proposed. However, the 2D COS did not give clear physicochemical differences between the three batters during mixing according to the authors [84]. The NIR results for the highly hydrated batter (56.7%) were difficult to analyse due to its high water content [20,21,84].

Rheological properties of dough are important for wheat quality characterization [80,84,85,86]. This research sought to obtain prediction models for rheological characteristics and to characterise the breadmaking quality of whole wheat using NIR spectroscopy, in order to offer a rapid tool to the farmers to know the quality of their product at the harvest moment. Tenacity (P), extensibility (L), deformation energy (W) and ratio P/L of dough were measured using traditional methods [86]. NIR spectra were acquired from these samples and models to predict the values from these parameters were developed. The SEC values achieved for the extensibility, deformation energy and tenacity of dough and the ratio between the two latter parameters were 5.27 mm, 9.97 × 10^−4^ J, 3.98 mm and 0.025 J mm^−1^, respectively. The four models were validated by cross-validation, and by independent validation. The precision obtained in these models was enough for being applied in harvesters or at delivering moment [86].

The different quality of rice in simulated cooking process was investigated by using scanning electron microscope (SEM), FTIR, XRD, and DSC methods [87]. The result shows that the starch granules of rice of low amylose content (AC) have more pores and cavities, which directly led to the swelling of rice starch granule [87]. With the change in temperature, the changes in intensity and width of the IR bands showed that the variation in proportion area of crystalline to non-crystalline was related to AC of rice. According to the interpretative sensitivity of the data obtained, XRD method was suggested adequate for characterizing the pasting property of different quality of rice. Besides, DSC method showed the AC was not relevant to pasting temperature and enthalpy [87].

The potential application of NIR, FT-Raman spectroscopy to monitor starch retrogradation in stored bread crumb was investigated [88]. Semolina-based bread was made and cut into slices, which were stored under controlled conditions in sealed plastic bags. The aging of the bread crumb was monitored by both NIR FT-Raman spectroscopy and a texture analysis over a period of 20 days. The use of 2D COS analysis in the spectral range of 390–975 cm^−1^ revealed characteristic differences among the spectra collected over time for bands that peaked at 480, 765, and 850 cm^−1^ [88]. The band at 480 cm^−1^ is studied here in detail. During the storage, the peak frequency of this band shifted towards lower wavenumbers, and its full width at half height decreased. Both of these parameters were highly correlated *R*^2^ of 0.92 and 0.95, respectively to crumb hardness measured by the texture analyser [88].

The gelation process of starch was monitored using IR spectra of starch in water while heating were obtained using ATR-MIR spectroscopy [89]. The authors evaluated the relationships between gelation and spectral changes using factor analysis, evolving factor analysis (EFA) and three-way PCA [89]. The authors reported that absorption values at 3300 and 1610 cm^−1^ decreased with temperature while absorptions at 1000 cm^−1^ increased [89]. The factor score plot patterns of amylose, amylopectin and rice starches were similar however those derived from potato and corn starches were unique [89]. van Velzen and co-workers [90] studied the factors associated with dough stickiness with the use of an ATR sampling module attached to a FTMIR instrument [90]. More recently Bock and collaborators [91] evaluated the use of ATR-MIR spectroscopy to monitor the addition of water on the gluten properties in dough [91,92]. The authors compared the OH stretch band of water in flour dough with that in H_2_O-D_2_O mixtures having the same water content revealed the formation of two distinct water populations in flour dough corresponding to IR absorption frequencies at 3600 and 3200 cm^–1^ [91,92]. The band intensity at 3200 cm^–1^, which is related to water bound to the dough matrix, decreased and shifted to lower frequencies with increasing moisture content of the dough [91,92]. The authors concluded that when bran is added to flour dough, water redistribution among dough components promotes partial dehydration of gluten and collapse of β-spirals into β-sheet structures [91,92]. According to the authors this transconformational changes could be related to the physical basis for the poor quality of bread dough containing added bran [91,92].

## 3. Conclusions

The measurement of starch chemical components (e.g., amylose and amylopectin), and physical characteristics of the endosperm such as granule type (A and B), and biophysical properties such as gelatinization, retrogradation, crystalinity were reported by several authors using either NIR or MIR spectroscopic. However, contradictory results in terms of precision and accuracy of the calibrations obtained were reported. Such differences were attributed to the accuracy of the reference method used (e.g., enzymatic or colorimetric methods), with interferences with other properties (e.g., lipids, proteins), range in composition, with the number of samples used to develop the calibration models, and the methods used (NIR, MIR, transmission, ATR, reflectance).

Infrared (IR) spectroscopy also has demonstrated its capability of in/on and at-line monitoring of different stages during wheat processing. This method is well adapted to identify and describe physical, chemical and physicochemical modifications occurring during wheat processing. Overall, the different data found in the literature demonstrated that the IR and in particular NIR spectroscopy can be used to generate relevant information to monitor wheat processing because it is the least perturbing method of exploring molecular changes, during the input of mechanical energy. It can be considered as a valuable method for the industry to analyse, control and understand the origin and construction of starch based products.

Overall, the main advantages of these techniques are the speed and ease of use in routine operations, determining potential savings, through reduction of analysis time and cost. However, the major disadvantage has been the difficulty of quantification and interpretation of data generated by spectroscopic methods. It is clear that the breadth of these applications, either in routine use or under developed is showing no sign of diminishing. The combination of these techniques with multivariate methods could be used as a tool on a large scale to be used in R & D or industrial applications.

However, the chemical or biophysical basis that determines the corner stone is still not well understood. The biggest challenges in the wider use of these technologies will be the interpretation of the complex data obtained. The interpretation of the models through multivariate methods in particular calibration development, the knowledge of the fundamentals of molecular spectroscopy in rheological analysis by IR spectroscopy is still not well known. The future development of these applications will provide the cereal and starch base food industry with very fast and non-destructive methods to quantify different samples providing the industry with a rapid means of qualitative rather than quantitative analysis.
